# Study on Carbonization Characteristics and Deterioration Mechanism of Recycled Concrete with Tailings and Polypropylene Fiber

**DOI:** 10.3390/polym14142758

**Published:** 2022-07-06

**Authors:** Tao Li, Meng Zhan, Xiuyun Chen, Fan Xu, Sheliang Wang, Xinxin Liu

**Affiliations:** 1 College of Architecture Engineering, Huanghuai University, Zhumadian 463000, China; zhanyi313@163.com; 2 College of Urban, Rural Planning and Architectural Engineering, Shangluo University, Shangluo 726000, China; liuxx2020@foxmail.com; 3 School of Civil and Architecture Engineering, Xi’an Technological University, Xi’an 710021, China; xufanxf1205@163.com; 4 School of Civil Engineering, Xi’an University of Architecture and Technology, Xi’an 710055, China; sheliangw@163.com

**Keywords:** polypropylene fiber, recycled concrete, tailings, carbonization characteristics, deterioration mechanism, micromorphology

## Abstract

To improve the deformation performance of recycled concrete with tailings (TRC), its carbonization characteristics and deterioration mechanism with different polypropylene fiber content were analyzed macroscopically and microscopically. The results showed that the fiber had little effect on the compressive strength, which increased first and then decreased, with the optimum content being 0.6%. The splitting tensile strength first increased and then tended to be stable, with the optimum dosage ranging from 0.6% to 0.9%. The more the content, the higher the peak strain and the lower the elastic modulus. The rising section of its constitutive curve changed little, while the falling section became more gentle. Carbonization made the relative dynamic elastic modulus change small with a trend of first increasing and then decreasing, and the optimum content was 0.6–0.9%. When the fiber content was small, the influence on the carbonization depth did not remain significant, but when it was large, the depth increased obviously, and this critical content was about 0.6%. Microscopically, through nuclear-magnetic resonance (NMR) and scanning electron microscope (SEM) analysis, due to the strong tensioning effect of the fiber, when a small amount was added, the porosity and pore structure had not been significantly changed, so the impact on its resistance to carbonization was not obvious. However, after excessive addition, the interface transition zone (ITZ) between different materials became larger, resulting in a significant increase of its harmful cracks and a great impact on the anti-carbonization ability, with the optimal content being about 0.6%. This study provides a theoretical reference for the deformation performance improvement measure of TRC, which would be helpful for the rapid promotion and application of green concrete in engineering practice.

## 1. Introduction

With the expansion of urbanization and the three-dimensional development of urban space, the infrastructure of China has also undergone earth-shaking changes. By the end of 2018, the housing construction area and highway mileage were 8.8 and 8.75 times the corresponding area in 2000 [1]. Faced with such a high growth rate, the demand for steel and concrete is also increasing day by day, which directly leads to the rapid accumulation of iron ore tailings (IOT) and the huge production of cement and sandstone materials, which then has a huge impact on the sustainable development of environment and economy. Compared with developed countries, the utilization rate of tailings and recycled aggregate in China has not reached 30%, which is far lower than 90% in developed countries [2], and has become the main restricting factor of our economic development [3]. It is an effective way to make TRC by combining the two together. The preliminary research results of our group proved that the appropriate amount of IOT can optimize the relevant mechanical properties of recycled aggregate concrete (RAC), but it would increase its brittleness [4,5]. Therefore, how to reduce its brittleness and enhance its deformation performance has become an urgent problem.

For fiber concrete, Meesala et al. [6] studied the effects of three different fiber types on the mechanical properties of RAC and natural aggregate concrete (NAC), whose results proved that fibers could significantly improve the mechanical properties of NAC and RAC. To improve the low strength of high-performance concrete, Maek et al. [7] made two kinds of recycled polypropylene fiber and mixed them into the concrete with the proportions of 0.5%, 1.0%, and 1.5% and studied their mechanical properties. The results showed that the optimal mechanical properties were achieved when the content of polypropylene fiber was 1.0%. Kazmi et al. [8] carried out the axial compression test on high volume fiber reinforced concrete columns and studied the axial stress–strain behavior of RAC with fiber, indicating that the addition of fiber increased the peak stress, peak strain, and ultimate strain, and then established the corresponding stress–strain curve model. Ahmad et al. [9] proved that when the nylon fiber content reached 1.5%, the mechanical strength of concrete increased significantly, then decreased gradually, and the durability parameters were significantly improved. Zaghloul et al. [10] and Fuseini et al. [11] used polyaniline nanofibers to make anti-corrosion coating and characterized its anti-corrosion protection by SEM, XRD, and other microscopic means. Luo et al. [12] introduced the influence factor of PVA to establish the carbon dioxide diffusion equation and determined its parameters according to the carbonization depth prediction model. Through the accelerated carbonization test, the correctness of the model and the numerical method is verified by comparing the experimental results with the numerical results. Wang et al. [13] discussed the synergistic effect of polypropylene fiber and rubber concrete from the aspects of mechanical properties, durability, and microstructure, the results showed that a large volume of polypropylene fiber (0.5%) could improve the drying shrinkage, ASR expansion, and frost resistance of rubber concrete. Cao et al. [14] studied the relationship between compressive strength and internal crack formation of composite-based cement fiber tailings by using an industrial CT system and SEM, which proved that the addition of fiber significantly improved the strength growth rate and the corresponding toughness.

For tailing concrete, Filho et al. [15] replaced natural fine aggregates with 10–80% of IOT to make concrete blocks. Through physical analysis, environmental analysis, and mechanical tests, the feasibility of the technology and environment was confirmed. MI Lvarez-Fernández et al. [16] designed the mix proportion of IOT, sand, and superplasticizer, then comprehensively considered the strength, workability, and constructability, which proved that it had high operability by the adding 20% tailings to the application. Oritola et al. [17] obtained five types of IOT from different sites, tested and evaluated them by using microscopic and physical examination techniques, whose results showed that IOT could be well used in concrete. To reduce the accumulation of tailings and reduce environmental pollution caused by cement production, Saedi et al. [18] reviewed the physical, chemical, and thermal activation methods that could improve the performance of tailings. Wei et al. [19] systematically studied the compressive and flexural mechanical properties of RAC with different tailing contents, and the results showed that when the tailing content was 30%, its mechanical properties were the best. Cui et al. [20] used the variance analysis method to define the compressive strength of TRC under different working conditions.

Presently, some scholars have made systematic research on RAC, tailing concrete, and fiber concrete separately, while studies on TRC with fiber are even rarer. Professor Wang Sheliang from Xi’an University of Architecture and Technology conducted a systematic macro and micro study on the carbonization [4,21], sulfate erosion [22], freeze-thaw cycles [23], and other characteristics of green concrete. Therefore, the carbonization durability and erosion characteristics of TRC are studied in this paper, which will contribute to its engineering application.

## 2. Experiment Material

Qinling ordinary Portland cement (p.o.42.5) and the sand in Bahe River were used. The natural coarse aggregate (NCA) was artificially crushed stone, while the recycled coarse aggregate (RCA) was produced by a factory in Xi’an, with particle size of 5–20 mm and continuous grading. The IOT comes from a tailings pond in Shangluo, Shaanxi Province. The gradation curve, main components, and physical performance indicators of the above-mentioned materials were detailed in my last article [4]. Polypropylene fiber used in the test was produced by a limited company in Xi’an, China, and its main physical properties are shown in Table 1.

To increase its fluidity, a polycarboxylic acid-based high-performance water reducer was used, which met the requirements of GB50119-2013 [24], and the main physical performance indicators are shown in Table 2.

## 3. Experiment Mix Ratio and Parameter Setting

Based on the previous research conclusions of our group on the mechanical properties and deformation properties of RAC [25,26] and TRC [4,21], the mixing proportion of RCA and IOT was set to 30%, and then different fiber mixing proportions (0.0%, 0.3%, 0.6%, 0.9%, and 1.2%) were considered. Referring to the relevant specifications [27,28,29] and references [30,31], the mixing proportion was designed. To facilitate comparative analysis, the water–binder ratio and the sand ratio of each mixing ratio were defined as 0.4 and 0.35, and the addition amount of water reducer was 0.3% of the total mass. When mixing, the fiber adopted the secondary stirring method. First, add water to mix half of the fiber to a uniform state, put them into the cement mixture, and mix, then add the fiber-water solution of the other half to a uniform state, and mix to the final state. After many attempts and microscopic tests, this method could maximize the fiber dispersion and optimize the corresponding mechanical properties of concrete. Through on-site trial and adjustment, its mixed proportion design under various working conditions is shown in Table 3.

The main procedures and parameters of carbonation test was shown as Table 4. The carbonization depth and the relative dynamic elastic modulus were measured as cubes (100 mm × 100 mm × 100 mm) and prisms (100 mm × 100 mm × 300 mm). The dynamic elastic modulus of carbonized 0 d, 7 d, 14 d, 28 d, 42 d, 56 d, 70 d, and 90 d was measured by Beijing Kangkerui NM-4A nonmetallic ultrasonic detection analyzer, while the carbonized depth of the cubes for 7 d, 14 d, 28 d, and 90 d were carried out by the phenolphthalein method. According to the requirements of the specification [32], the corresponding test block was taken out and cracked at the corresponding age, and the crushed powder on the surface of the test block was brushed off, and the section was sprayed with a concentration of 1% phenolphthalein alcohol solution. After 30 s, the digital display carbonization depth tester (accurate to 0.01 mm) was used to measure the depth at measuring points every 10 mm of the original carbonization surface, and the average carbonization depth was taken as the carbonization depth under this carbonization age.

## 4. Experiment Conclusion

### 4.1. Cube Compressive Strength

**Figure 1:**
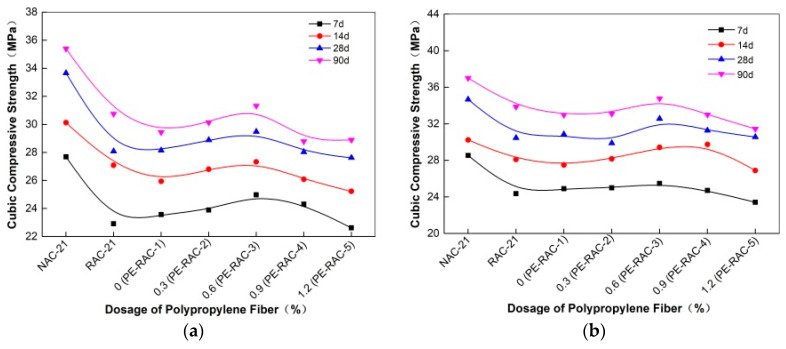
Effect of fiber on cube compressive strength before and after carbonization: (**a**) before carbonization; (**b**) after carbonization.

**Figure 2:**
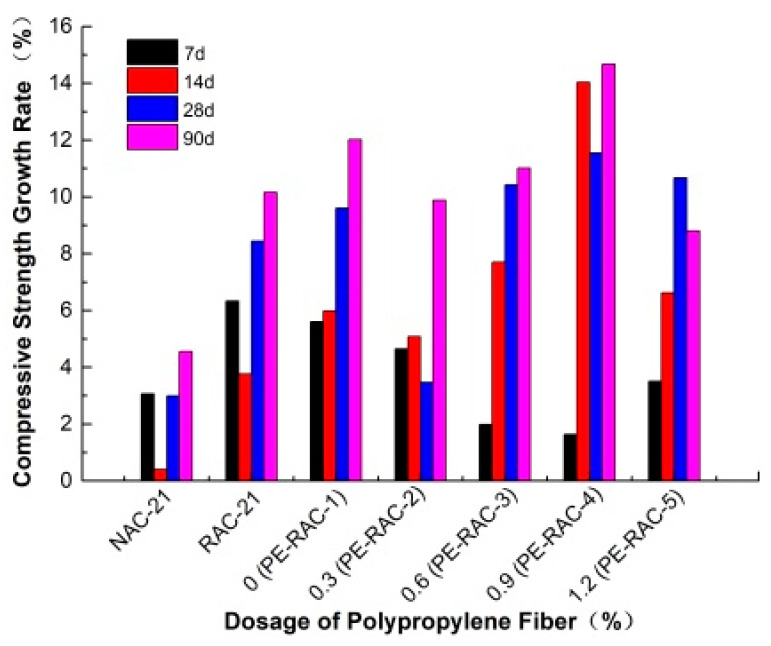
Growth rate of compressive strength before and after carbonization.

The influence of fiber content before and after carbonization on the cube compressive strength of TRC is shown in Figure 1, and Figure 2 illustrates the strength growth value of each age. It can be seen that the influence of polypropylene fiber on the cube’s compressive strength was not obvious. When the content was low (<0.6%), as the fiber content increased, the strength value before and after carbonization indicated a slowly increasing trend. While the content was high, the opposite phenomenon appeared, but the overall change was small. For example, compared with the content of 0.0% (PE-RAC-1), when the dosage reached 0.6 (PE-RAC-3), the strength value of each age before carbonization increased by 5.96%, 5.37%, 4.73%, and 6.40%, respectively, and compared with 2.32%, 7.08%, 5.52% and 5.44% after carbonization. Wang et al. [13] and Eaha et al. [33] obtained similar rules, while Akca et al. [34] and Yuan et al. [35] proved that fibers had little effect on cube compressive strength. Figure 1 also revealed that the addition of polypropylene fiber could not improve the cube compressive strength of TRC in essence, whose intuitive performance was that the value of different mixing conditions under most ages still owned lower than RAC (RAC-21) after the addition. Carbonization slowed down the strength difference and made the curve at the same age became mild. According to Figure 2, the growth rate value demonstrated irregular changes before and after carbonization. Therefore, it was necessary to study its deterioration mechanism by microscopic means.

### 4.2. Splitting Tensile Strength

**Figure 3:**
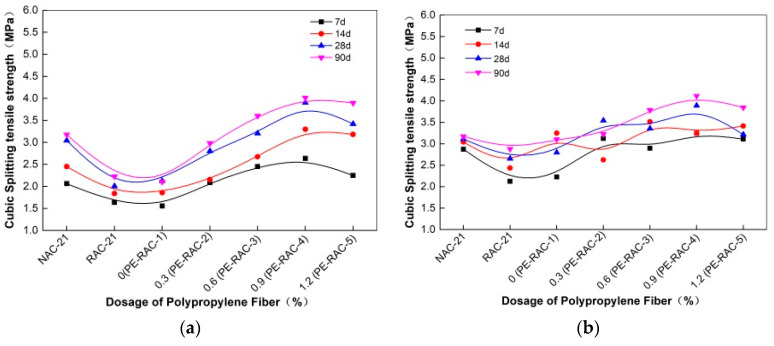
Effect of fiber on splitting tensile strength before and after carbonization: (**a**) before carbonization; (**b**) after carbonization.

Figure 3 displays the variation law and correlation curve of cube splitting tensile strength of TRC before and after carbonization. It can be seen from Figure 3a that the splitting tensile strength of TRC at different curing ages (7 d, 14 d, 28 d, and 90 d) increased with an appropriate amount of fiber, and the increasing effect was obvious. When the content increased from 0% (PE-RAC-1) to 0.9% (PE-RAC-4), the strength also increased by 57.56%, 44.01%, 50.82%, and 71.10%. Conversely, when it increased from 0.9% (PE-RAC-4) to 1.2% (PE-RAC-5), the strength decreased by 14.6%, 3.56%, 12.42%, and 2.96%, which implied that the excessive fiber content made its properties decline. According to Figure 3b, carbonization made the values fluctuate in varying degrees, and the fluctuation trend was consistent with that before carbonization, that was, it added the fluctuation before the optimum dosage (0.6–0.9%), and after the optimum dosage, it decreased in varying degrees. Concurrently, it can also be seen that carbonization reduced the strength difference at each age and made the change curve closely. Through the research on the quasi-static and dynamic properties of high-strength concrete, Hui et al. [36] obtained a conclusion similar to that in the article, and the optimum content of fiber was 0.12%. Wan et al. [37] studied the properties of fiber reinforced geopolymer concrete and obtained that the optimum content of fiber was 1.0%.

**Figure 4:**
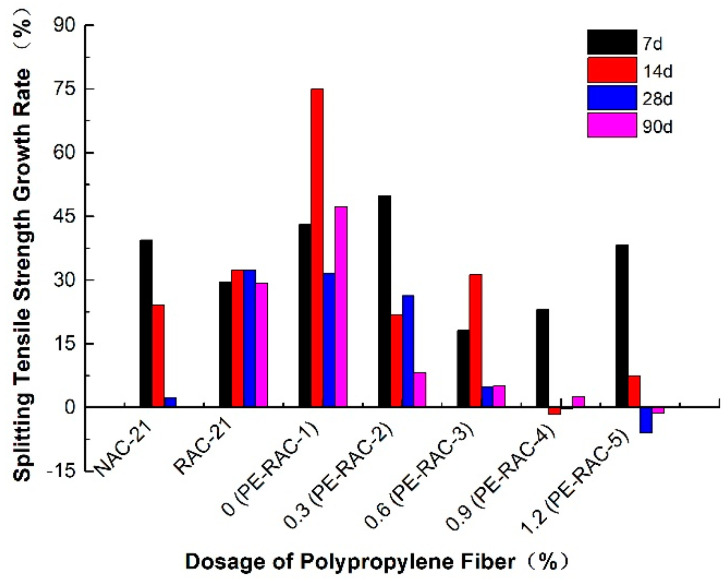
Growth rate of splitting tensile strength before and after carbonization.

According to Figure 4, carbonization made the strength value fluctuate irregularly at each age, however, when the content was higher (0.9% and 1.2%), the excess fiber made the internal accumulation of the concrete and the bond with the concrete became weaker. Contrarily, some tensile strength values were slightly reduced. Finally, it can also be obtained from the above figures that fiber had a significant effect on the improvement of the tensile performance of TRC, and its tensile strength can be better than that of NAC at some dosage, excessive mixing can also degrade its performance. Therefore, the actual amount of fiber incorporation should be strictly controlled in future engineering practice.

### 4.3. Axial Compressive Strength

**Figure 5:**
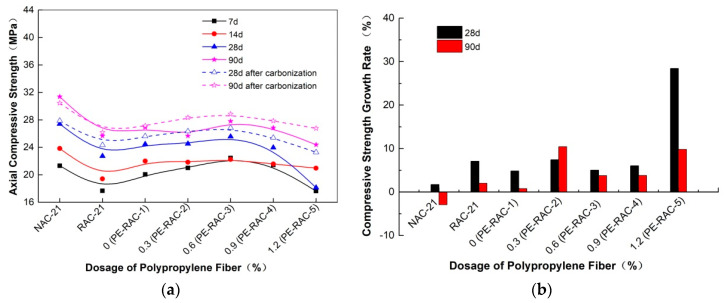
Effect of fiber on axial compressive strength before and after carbonization: (**a**) axial compressive strength; (**b**) growth rate of axial compressive strength.

Figure 5 exhibited the effect of fiber on the axial compressive strength of TRC before and after carbonization. It can be seen that the influence law of fiber on axial compressive strength was also similar. With the increase of fiber content, it increased first and then decreased, but the change trend was small. According to Figure 5a, when the fiber content was 0.6%, the axial compressive strength reached the maximum, whose results were similar to that of Xu et al. [38]. Compared with 0% (PE-RAC-1), the strength values of 7 d, 14 d, 28 d, and 90 d only increased by 11.9%, 1.09%, 4.54%, and 3.23%. Compared with NAC, it increased by 5.27% at 7 d and decreased by 6.65%, 6.71%, and 11.39% at 14 d, 28 d, and 90 d, which also demonstrated that the reinforcement effect of fiber on its strength value was limited. The dotted line in the figure showed the variation law of axial compressive strength at 28 d and 90 d of carbonization, which was consistent with that before carbonization. It can be seen from Figure 5b that, except for PE-RAC-5 at 28 days of carbonization, the growth rates of other strengths were all below 10%, which also indicated that carbonization had a weaker influence on it.

### 4.4. Deformation Capacity

**Figure 6:**
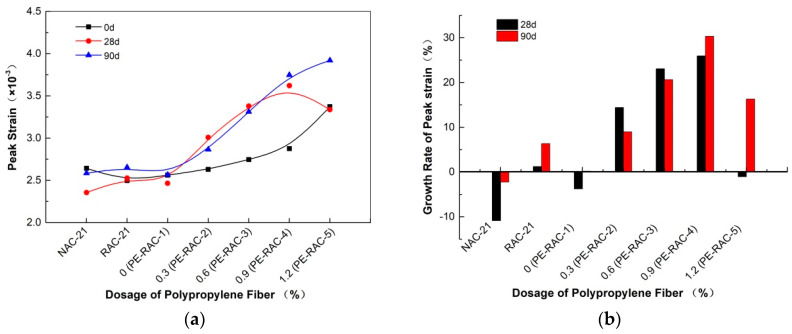
Effect of carbonization on peak strain: (**a**) change curve of peak strain; (**b**) change rate of peak strain.

**Figure 7:**
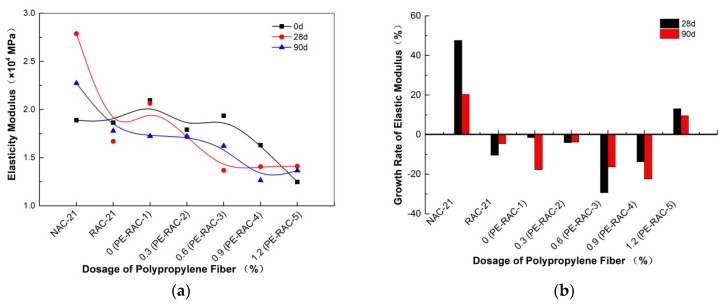
Effect of carbonization on elastic modulus: (**a**) change curve of elastic modulus; (**b**) change rate of elastic modulus.

Figure 6 and Figure 7 show the influence of different fiber content on the deformation performance of concrete under different carbonization ages. It can be seen that at different carbonization ages, due to its excellent tensioning effect, the higher the content was, the greater the peak strain was, and the more the elastic modulus decreased. As can be seen from Figure 6, the peak strain curve generally revealed an increasing trend with the carbonization cycle. This phenomenon was similar to that observed in the literature [39,40]. For example, when the fiber content was 0.9% (PERAC-4), the obtained carbonizations for 28 d and 90 d were 25.94% and 30.32% higher than that for 0 d. When the content was high, the peak strain growth trend slowed down or even decreased due to its stacking and overlapping effect. When the content was 1.2% (PE-RAC-5), the carbonization for 28 d and 90 d increased by −1.04% and 16.28%, compared with that for 0 d. 

As can be seen from Figure 7, carbonization made the elastic modulus of concrete fluctuate in varying degrees with different fiber content. In the case of non-carbonization, when the content was small (<0.6%), the elastic modulus hardly changed, with approximately horizontal, and when the content was large (>0.6%), it had a downward trend. When the content was high (>0.6%), the decreasing trend appeared. As the carbonization cycle became longer, the elastic modulus fluctuation trend of the concrete with a larger amount was weakened and gradually became flat. Similar results were also observed by Wang et al. [40] who also established their corresponding quantitative expression relationship.

### 4.5. Stress–Strain Constitutive Curve

**Figure 8:**
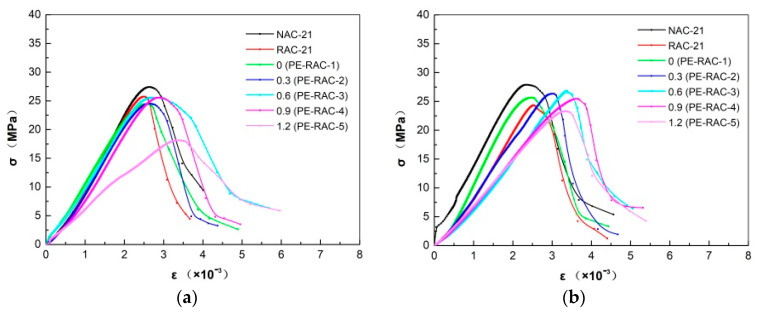

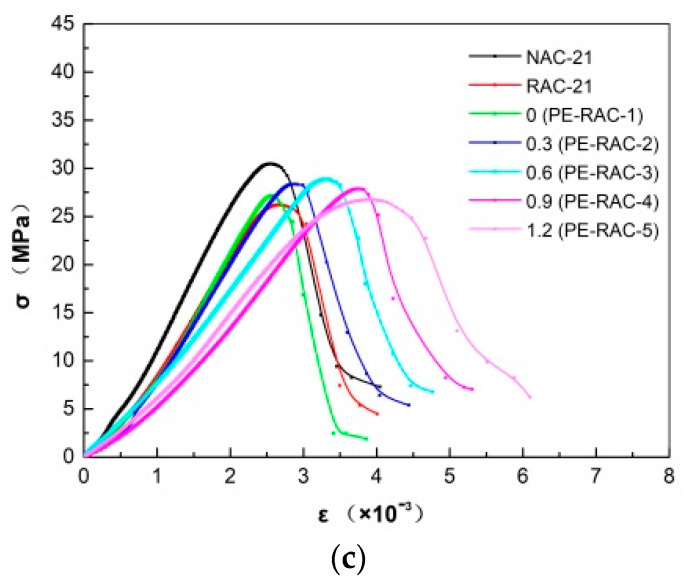
Effect of carbonization on stress–strain curve: (**a**) carbonization for 0 d; (**b**) carbonization for 28 d; (**c**) carbonization for 90 d.

Figure 8 manifested the changes in the stress–strain curve of concrete at various carbonization erosion ages. It can be seen that the addition of fiber made the stress at its peak point change little, but the strain at the peak point changed greatly, whose intuitive result was that the slope of the rising line of the constitutive curve decreased gradually, and the larger the approximate content, the smaller the slope. Similar to the influence law of adding tailings [4], the longer the carbonization cycle, the more similar the change law of the descending section, and the slower the stress decay rate, the larger the area enclosed by the stress–strain curve and its projection on the horizontal axis, that is to say, the energy dissipation capacity of the experiment block was gradually enhanced.

## 5. Durability Performance

The design and calculation of dynamic elastic modulus and carbonization depth were carried out in accordance with the requirements of the specification [32]. To facilitate comparison and analysis, the relative dynamic elastic modulus was used as the evaluation index in the article, and its calculation formula was as follows:

(1) $$P_{n}=\frac{E_{dn}}{E_{d0}}\times 100=\frac{f_{n}^{2}}{f_{0}^{2}}\times 100$$

(2) $$\bar{d_{i}}=\frac{1}{n}\sum_{i=1}^{n}d_{i}$$

where *P_n_* is the relative dynamic elastic modulus of the *i*-th concrete test block after the corresponding carbonization age. *f_n_* (*f*_0_) is the fundamental frequency of the non-carbonized surface of the *i*-th test block after carbonization for the *n*-th (0-th) day (Hz). *d_i_* is the carbonization depth of the *i*-th measuring point, and *n* is the total number of measuring points. In case of coarse aggregate blocking at the measuring point or cross coupling carbonization (edge carbonization) around the carbonization surface, the point with small fluctuation shall be selected around the corresponding point.

**Figure 9:**
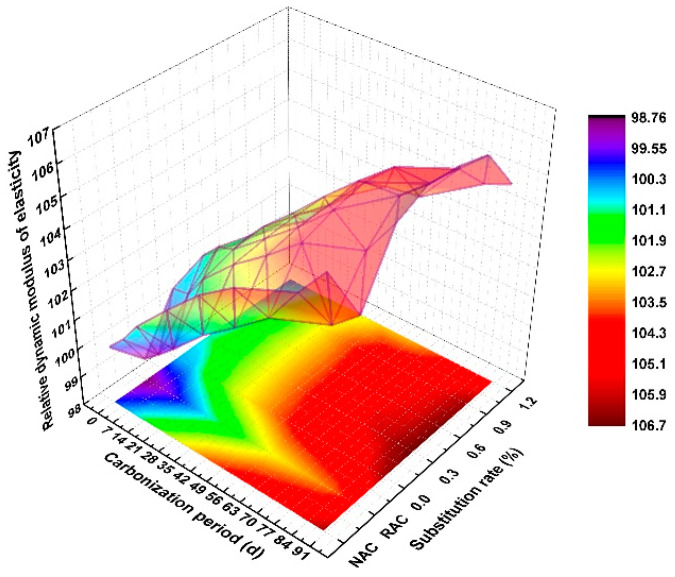
Relative dynamic elastic modulus.

As can be seen from Figure 9, with the increase in carbonization age, the relative dynamic elastic modulus displayed an increasing trend, but the effect of the increase was relatively limited. When carbonized for 90 days and 0 d, the maximum dynamic elastic modulus occurred at the fiber content of 0.6%, which increased by 6.56% and 5.83%, compared with that of NAC at a similar carbonization age. Meanwhile, the projection on the XY plane and the related equipotential curve indicated that the relative dynamic elastic modulus of RAC was the lowest under the same carbonization age, which also proved that the quality of the matrix structure was the main factor that determined the carbonization dynamic elastic modulus. Under the same carbonization age, the relative dynamic elastic modulus increased first and then decreased after the fiber addition, and the maximum occurred when the content was 0.6–0.9%. However, the addition of excess fiber made its value attenuation more moderate. For example, when carbonized for 28 d, the fiber increased from 0.3% to 1.2%, and the value of relative dynamic elastic modulus decreased from 102.67 to 102.21.

**Figure 10:**
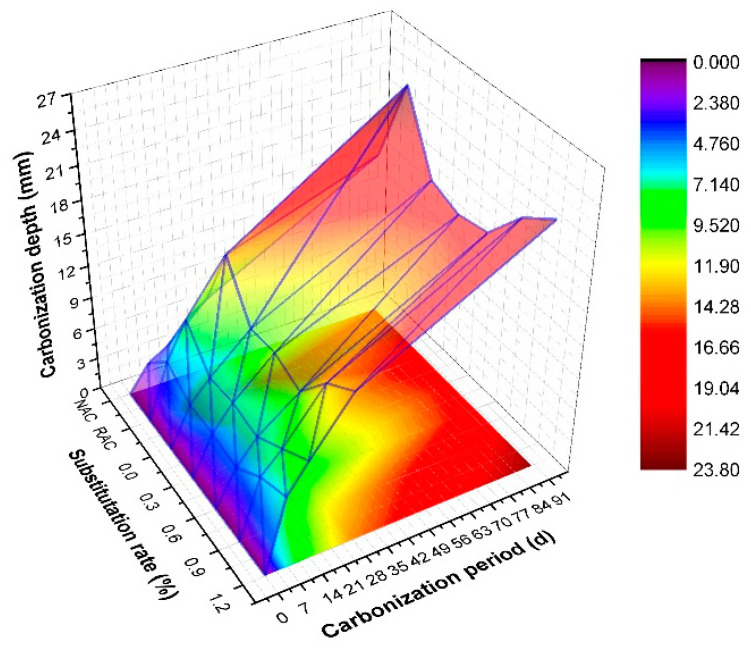
Carbonization depth.

From Figure 10, under the same mixing ratio, the longer the carbonization period was, the greater the carbonization depth was. The porosity of RAC increased significantly due to the existence of mortar particles on the surface of RCA, ITZ, brick particles, and cracks during aggregate crushing. Compared with NAC, the carbonization resistance of RAC decreased sharply and the carbonization depth increased significantly, for example, when RAC-21 was carbonized for 7 d, 14 d, 28 d, and 90 d, its carbonization depth increased by 111.74%, 111.39%, 96.36%, and 52.78%. Polypropylene fiber had excellent ductility and ultimate elongation and formed a certain grip force with concrete slurry. Due to the increase of ITZ between different materials, it was easier to produce micro-cracks on the contact surface. When the fiber content was low (≤0.6%), the overlapping effect between different fibers was also weak, so it had little effect on its overall carbonization resistance, for example, at each carbonization age (7 d, 14 d, 28 d, 90 d), when the fiber content increased from 0% (PE-RAC-1) to 0.6% (PE-RAC-3), the carbonization depth also increased by 10.24%, 2.12%, 7.05% and 2.09%, with relatively little influence. When the content was large, on the one hand, the ITZ between fiber and concrete increased greatly, on the other hand, the overlapping effect between fibers in different directions was also obvious, and these overlapping zones were the cross weak areas inside the concrete, which would further increase the porosity of TRC and significantly enhance its carbonization depth. When the fiber content increased from 0.6% (PE-RAC-3) to 1.2% (PE-RAC-5), the carbonization depth increased by 100.90%, 66.30%, 66.55%, and 33.07% at different carbonization ages, whose degradation effect on its carbonization resistance was quite obvious. This phenomenon was also observed in the literature [21,41], which showed that the carbonization depth and carbonization rate coefficient first decreased and then increased with the fiber content, and the optimum content was 1.5% (steel fiber) and 1.0% (carbon fiber).

## 6. Micro-Morphological Analysis

The microstructure of concrete directly affects its macro mechanical properties and durability [42]. Therefore, the research on the microstructure and pore distribution, that is, the discussion on the deterioration mechanism, has become an important means to better and more truly explain the macro phenomenon [43].

### 6.1. Nuclear-Magnetic Resonance (NMR)

The X-coordinate in the T_2_ distribution spectrum was related to the pore size of porous materials. The larger the pore size, the greater the freedom degree of pore water and the longer the relaxation time. The Y-coordinate was the signal intensity, and its amplitude reflected the number of pores, the larger the amplitude, the greater the number of pores [44]. The T_2_ relaxation curve was usually a multi-peak curve. The position of each peak corresponded to pore size, whose peak area was the definite integral of the T_2_ curve, and its value represented directly the pores’ number [45]. The change of peak area could also characterize the change of total porosity in porous materials. Therefore, NMR technology can reflect the porosity, pore size, pore number, and other pore characteristics of porous materials [46].

**Figure 11:**
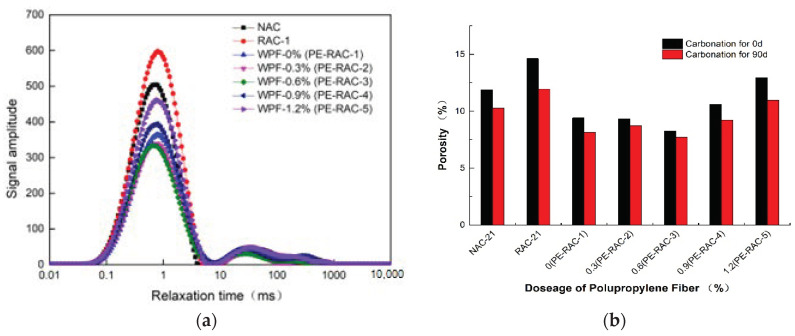
Porosity test results: (**a**) T2 relaxation curve (0 d); (**b**) porosity.

Figure 11 exhibits the porosity test results of TRC with different fiber content before and after carbonization. It can be seen that, except for PE-RAC-5 with six spectral peaks, the other concrete had three spectral peaks, which also indicated that when the fiber content was high (1.2%), mainly due to its unique spatial network structure, large pore diameter was generated. The longitudinal axis represented the pore numbers, and the position of the first wave peak had the largest amplitude, indicating that the pores’ numbers with relaxation time in this range was the largest.

**Figure 12:**
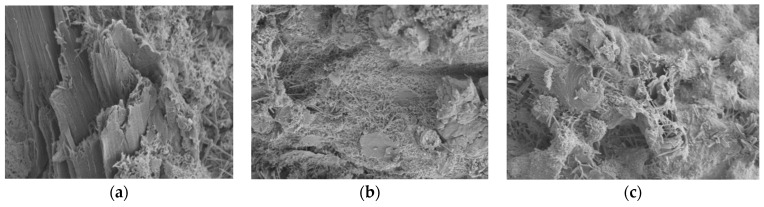
Microscopic morphology diagram of TRC with different fiber content before carbonization: (**a**) 0% (PE-RAC-1); (**b**) 0.6% (PE-RAC-3); (**c**) 1.2% (PE-RAC-5).

Based on the NMR results, the NMR signal quantity per unit volume was proportional to the porosity, and the values before and after carbonization could be obtained, as shown in Figure 12b. When the initial porosity was large, carbonization could significantly promote the hydration of concrete. For example, the porosity of RAC-21 and PE-RAC-5 was reduced by 18.32% and 15.43% after carbonization respectively. When the fiber content is low, the effect on the porosity is different. For example, for PE-RAC-3, the porosity carbonization for 90 d was only 6.42% lower than the initial porosity. In terms of pore structure, compared with the concrete with 0% content (PE-RAC-1), the maximum amplitude increased by −7.02%, −8.22%, −7.96%, and 26.51% with the increase of fiber content, and the corresponding first peak area changed by −6.73%, −7.21%, −10.78%, and 28.29%, respectively, which indicated that when the content was small (≤0.6%), the fiber could reduce the pore numbers to a certain degree, but when it was large, it increased the pore numbers to a large extent.

### 6.2. Scanning Electron Microscope (SEM)

**Figure 13:**
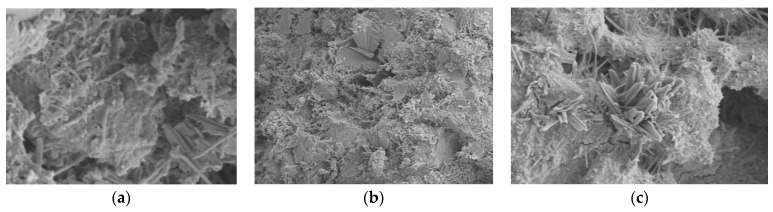
Microscopic morphology diagram after carbonization for 90 d: (**a**) 0% (PE-RAC-1); (**b**) 0.6% (PE-RAC-3); (**c**) 1.2% (PE-RAC-5).

Figure 12 and Figure 13 represent the microscopic morphology of TRC with the fiber content of 0.0% (PE-RAC-1), 0.6% (PE-RAC-3), and 1.2% (PE-RAC-5) at carbonization for 0 d and 90 d. In terms of chemical composition, the figures revealed the existence of needle-like, rod-like, and net-like ettringite, but there was no obvious difference in content. This was mainly because of the existence of IOT with 30% promoting the secondary hydration of cementitious materials. It also proved that the fiber had no active function and did not promote the hydration of cement. From the matrix structure, when the content was low, there was a large number of C-S-H gels on the surface of the fibers, which could enhance the bond strength between the fiber and the concrete matrix, and the fiber’s three-dimensional spatial network structure distribution system and its viscous effect strengthened the bonding between the matrix structure and the holes, cracks, and the un-hydrated parts in the concrete, thereby enhancing the integrity of the matrix structure. However, it can also be seen that there were many cracks and holes in the ITZ between fiber and cement paste. On the one hand, due to the unique distribution mode of fiber in concrete, the fibers in different directions intersected and overlapped, and the increase of contact area between different materials would directly or indirectly increase the porosity of the matrix structure. On the other hand, under the action of external force, in the process of fiber pulling out, breaking, or thermal expansion and cold contraction, with the generation of deformation, there would be stress concentration in the ITZ around the fiber, as well as cracks or pores around the fiber. When the fiber content was small, this effect was relatively weak.

Comparing Figure 12 with Figure 13, it can be seen that there were micro-cracks around the waste fibers in the matrix structure before and after carbonization. This was mainly due to the three-dimensional irregular distribution of waste fibers in the concrete, and the staggered overlapping of fibers in different directions, which made the contact area between different materials and the micro-cracks increase in the concrete. For the hydration-promoting effect of carbonization, the concrete developed more perfectly after carbonization, and it was intuitively shown as the increase of needle or rod.

In general, the fiber had positive and negative influences on the matrix structure, and the two worked together to produce the above-mentioned main mechanical and durability change characteristics, which had a greater impact on the tensile and deformation properties. However, when the content was large, its negative effect increased significantly. From the perspective of the text, the optimal dosage was 0.6%.

## 7. Conclusions

Through the analysis of the carbonization characteristics and deterioration mechanism of TRC with different carbonization cycles and fiber contents, the following conclusions can be drawn:

(1) Polypropylene fiber had little effect on cube compressive strength and axial compressive strength, showing a trend of increasing first and then decreasing, with the best content of 0.6%. The splitting tensile strength increased obviously, increased first, and then tended to be stable, and the optimum content was 0.6–0.9%. The higher the content was, the greater the peak strain. The elastic modulus fluctuated slightly, the greater the content, the lower the elastic modulus. It can optimize the ductility of the prismatic test block and reduce the fluctuation of the falling section of the constitutive curve. The situation after carbonization was similar to that before carbonization, the position of certain peak points was approximately unchanged, and made their corresponding values fluctuate less.
(2) Carbonization made the relative dynamic elastic modulus change less. As the fiber content increased, its value approximately increased first and then decreased, with a peak content of 0.6–0.9%. When the content was small, it had little effect on the carbonization depth, when it was large, the carbonization depth increased greatly and the carbonization resistance deteriorated seriously. The content in this critical state obtained approximately 0.6%.
(3) Through NMR and SEM analysis, the fiber had a strong pulling effect, a small amount of addition can greatly improve its deformation ability, and had little effect on the pore diameter and porosity. However, after excessive addition, the contact area between different materials would increase. At the same time, the stress concentration phenomenon of the fiber after being stressed would also cause the harmful gaps in the concrete to increase, reducing the anti-carbonization ability, and the optimum mixing amount was approximately 0.6%.

Through macro and microanalysis, when the content of polypropylene fiber was 0.6%, its porosity and pore structure reached the optimum. At this time, TRC can not only obtain the best mechanical and deformation properties but also its resistance to CO_2_ corrosion is not degraded.

## Figures and Tables

**Table 1:** Basic performance parameters of polypropylene fiber.

Density (kg/cm^3^)	Length (mm)	Equivalent Diameter (mm)	Eensile Strength (MPa)	Breaking Elongation (%)	Elastic Modulus (MPa)	Retention Rate of Alkali Resistant (%)
1.12	22	0.08	>350	12–40	>4000	>94.4

**Table 2:** Main physical properties of water reducer.

Varieties	Density (g/m^3^)	pH Value	Water Solubility	Solid Content (%)	Cl^−^ CONTENT (%)	Na_2_SO_4_ Content (%)	R_2_O Content (%)
CLB-61	1.05 ± 0.02	6~7	Mutually dissolvable	20.0 ± 1.0	≤0.1	≤2.0	≤5.0

**Table 3:** Mix proportion design under different working conditions (kg/m^3^).

Test Block Number	Cementitious Material	Coarse Aggregate		Fine Aggregate		Water	Fibers	Water Reducer
		NCA	RCA	Sand	IOT			
NAC-21	538	1063	0	572	0	215	0	0
RAC-21	538	735	315	566	0	215	0	0
PE-RAC-1	538	748	320	403	173	215	0	8.07
PE-RAC-2	538	748	320	403	173	215	1.614	8.07
PE-RAC-3	538	748	320	403	172	215	3.228	8.07
PE-RAC-4	538	748	320	403	172	215	4.842	8.07
PE-RAC-5	538	748	320	403	172	215	6.416	8.07

**Table 4:** Experiment procedures and main parameters.

Sequence of Steps	Making and Curing	Drying	Covering	Carbonizing	Loading and Testing
Parameter setting	Standard curing for 28 d	Drying for 48 h at 60 °C	Covering 5 surfaces with paraffin and leaving a carbonized surface	CO_2_ levels: (20 ± 3)% Temperature: (20 ± 2) °C Humidity: (60 ± 5)%	Loading rate: 0.5 MPa/s (compressive strength) 0.05 MPa/s (tensile strength)
